# Genome-Wide Analysis, Identification, and Transcriptional Profile of the Response to Abiotic Stress of the *Purple Acid Phosphatases* (*PAP*) Gene Family in Apple

**DOI:** 10.3390/ijms26031011

**Published:** 2025-01-24

**Authors:** Hong-Chao Liu, Lei Rao, Jia-Hui Meng, Wen-Teng Zuo, Ting-Ting Sun

**Affiliations:** 1 College of Horticulture, China Agricultural University, Beijing 100193, China; liu1413325675@163.com; 2 State Key Laboratory of Tree Genetics and Breeding, Key Laboratory of Tree Breeding and Cultivation of the State Forestry Administration, Research Institute of Forestry, Chinese Academy of Forestry, Beijing 100091, China; rl241215@163.com (L.R.); jocelyns_email@163.com (J.-H.M.); zuowt0102@163.com (W.-T.Z.)

**Keywords:** apple, purple acid phosphatases, gene expression, phosphorus stress, drought stress

## Abstract

Purple acid phosphatases (PAPs) play a significant role in plant phosphorus nutrition and can not only release phosphorus from the soil but also regulate the distribution of phosphorus in plants throughout their entire growth and development process. Moreover, members of the PAP protein family exert a more extensive influence on plant mineral homeostasis, developmental processes, and stress responses. Three clusters of purple acid phosphatases, including 31 putative genes, were identified in apples (*Malus domestica*) by searching the Genome Database for Rosaceae. The structure, chromosomal distribution and location, phylogeny, motifs, and cis-acting elements in the gene promoter regions of the *MdPAP* gene family are reviewed. These genes exhibit different expression patterns in different tissues. For example, almost all *MdPAP* genes are strongly expressed in the roots, except for *MdPAP10*, *MdPAP12*, and *MdPAP27*. Similarily, all *MdPAPs* were expressed in the leaves while the transcript levels of *MdPAP7*, *MdPAP10*, *MdPAP15*, *MdPAP21*, *MdPAP24*, *MdPAP26*, *MdPAP29*, and *MdPAP30* were highest in apple flowers. Overall, the expression of the 31 genes significantly changed in either the roots or leaves following the application of phosphorus and/or drought stress. These results indicate that MdPAP family members play a role in plant adaptation to adverse environments. This work explores the adaptative responses to phosphorus and/or drought conditions in apple and establishes a foundation for an enhanced comprehension of the evolution of PAP families and the exploration of the genes of interest.

## 1. Introduction

Phosphorus (P) serves as a crucial nutrient that is indispensable for the growth and development of plants. The primary form of phosphorus that plants assimilate from the soil is inorganic phosphate (Pi) [1,2]. Pi is integral to all major metabolic processes in plants, including photosynthesis and respiration. Furthermore, it functions as a crucial structural component of essential biomolecules, such as ATP, NADPH, phospholipids, nucleic acids, and sugar-phosphates. However, the concentration of Pi in most soils is significantly lower than what is required for optimal plant growth. This is primarily because most P exists in the form of organophosphate, which cannot be directly absorbed by plants [3].

In response to phosphorus deficiency, plants have developed a sophisticated array of adaptation strategies to increase their acquisition and utilization of phosphorus. A key mechanism involves the synthesis and secretion of acid phosphatases (APases). Since organophosphorus is difficult for plants to use directly, only the Pi released after acid phosphatase degradation can be absorbed by plant roots [4,5]. Within the APase family, purple acid phosphatases (PAPs) are classified under the metallophosphoesterase superfamily, which also encompasses phosphoprotein phosphatases and exonucleases. PAP exhibits distinct biochemical and molecular properties, including a purple or pink hue in the enzyme solution upon extraction, resistance to tartaric acid inhibition, a binuclear metal center, and five conserved motif blocks within its amino acid sequence [6]. Eukaryotic PAPs are categorized into two distinct groups: high-molecular-weight enzymes, with a mass of 55 kDa, and low-molecular-weight enzymes, with a mass of 35 kDa. All PAPs contain five blocks of conserved metal ligating residues; however, the quantity, position, and identity of these residues vary among different family groups [7,8]. Members of the PAP family possess seven metal ligating residues, denoted DxG–GDXXY–GNH(D/E)–VXXH–GHXH. These seven conserved amino acid residues play crucial roles in the formation of Fe^3+^-X (X represents bivalent metal ion Fe^2+^, Mn^2+^, or Zn^2+^) bimetallic ion catalytic centers [5,9]. Plant PAPs have been systematically classified into three principal groups, as determined by their predicted protein sequences [7]. These groups are as follows: Groups I and II, which consist of oligomeric PAPs with high molecular weights, and Group III, which is composed of mammalian-like low-molecular-weight monomeric PAPs [5,7].

PAP is an intracellular and secretory acid phosphatase isoenzyme that is upregulated after phosphorus deficiency in plants. Biochemical signatures have been analyzed across various plant species, revealing that *Arabidopsis* contains 29 members of the *PAP* gene family [5,10,11], tomato has 25 PAP members [12,13], 29 *LaPAPs* have been identified in lupin [8,14], 26 members have been identified in rice [15], 35 members have been identified in soybean [16], 33 members have been identified in maize [17], 25 putative PAPs have been identified in chickpea [18], 25 putative *Jatropha curcas PAP* genes have been identified [19], 19 *CsPAP* members have been identified in tea [20], and 105 *TaPAP* genes have been identified in the hexaploid nature of wheat [21].

Plant PAPs play multiple roles throughout plant growth and development. The overexpression of *AtPAP2* in *Camelina sativa* [22], potato [23], and *Arabidopsis* [24] resulted in faster plant growth. *AtPAP10* is a predominantly secreted APase that is primarily localized to the root surface [25]. Analysis of *atpap10* mutant strains and overexpression strains revealed that *AtPAP10* plays a vital role in the utilization of exogenous organophosphorus [25,26,27]. Overexpressing *OsPAP10c* promoter improved crop production [28]. Plant PAPs also play important roles in cell wall biosynthesis, flower development, root growth, symbiotic associations, carbon metabolism, phospholipid hydrolysis, defense responses, and cell signaling [29].

PAPs are involved in the acquisition and utilization of Pi and in the response to abiotic stresses. Upon Pi starvation, the transcript and corresponding protein levels of PAP are highly induced in plants. Many PAPs improve low phosphorus tolerance when overexpressed. Overexpressing *AtPAP15* enhances phosphorus efficiency and increases plant dry weight and phosphorus content in soybean [30]. The transcription of *AtPAP12* is induced by Pi starvation, and overexpressing *AtPAP12* improves low-Pi tolerance [31]. The expression of *AtPAP17* was upregulated during Pi deprivation, and the *AtPAP17*-overexpressing plants presented greater Pi absorption and APase activity than the WT plants under Pi-sufficient conditions [32]. The expression of most *BnaPAPs* in *Brassica napuswere* was significantly induced by low-P conditions [33]. The overexpression of *GmPAP3* has been found to increase soybean resilience to oxidative damage under conditions of salinity and osmotic stress. Additionally, PAPs also play a role in the metabolism of ROS [34], nitrogen fixation, cellulose [35], anthocyanin accumulation [36], phytate accumulation [18], and carbon metabolism [23]. *GmPAP17* overexpression increased the acid phosphatase activity and growth performance of hairy roots under phosphorus deficiency [37]. *TaPAPs* respond to abiotic stresses, including low temperatures, drought, and anaerobic conditions [21]. The expression of *StPAP* in potato changed in response to various phosphorus levels, phosphorus-solubilizing bacteria, and freezing stress, drought, heat, darkness, and N/K/Ca/Fe/Mg/Zn deficiency stresses [29].

Apple (*Malus domestica*) is an economically important fruit that is widely cultivated in the worldwide. However, the external environmental stress, such as drought stress, nutrient element deficiency, excess of nutrient elements, and multiple environmental stresses, affects the normal growth and quality of apple. *PAP* genes play important roles in plant stress resistance, so understanding the function of *PAP* gene in apple is essential. The apple genome sequence has provided new tools to facilitate the discovery of genes [38] and other functional elements in apple [39]. This provides robust resources and instruments for the identification and examination of the *PAP* gene family on a genome-wide scale.

We performed a comprehensive genome-wide identification of 31 *PAP* genes in *Malus*, analyzed their predicted chromosomal locations, examined their gene structures, phylogenetic relationships, conserved domains, gene structures, cis-elements, and monitored their patterns of transcription in different plant parts. Additionally, we monitored patterns of transcription under various conditions and in response to diverse environmental stresses. The purpose of this study was to lay a foundation for the further functional analysis of *PAPs* and to enable the application of genetic engineering methods to improve P uptake and utilization efficiency in apple.

## 2. Results

### 2.1. Genome-Wide Identification and Chromosomal Distribution of MdPAP Genes in Apple

To validate the presence of *PAP* genes in apple, protein sequences from rice, *Arabidopsis*, and poplar that were previously extensively searched in the apple genomics database were used. A total of 38 potential candidates were identified in *Malus*. These included MD02G1021200 and MD02G1021400 on chr2; MD03G1203900 and MD03G1203400 on chr3; MD04G1009700 and MD04G1009800 on chr4; and MD07G1029700, MD07G1028100, MD07G1028400, and MD07G1028500 on chr7. The four gene pairs were proximately located on their respective chromosomes, and the protein sequences derived from each pair exhibited significant similarity. This led us to hypothesize that these genes were tandem duplicates. Consequently, each pair was considered a single complete PAP sequence. A total of 31 *PHT* genes were identified in apple. Table 1 provides detailed information for each gene, including its name, genome position, open reading frame (ORF) length, number of exons, molecular weight (*M_W_*), isoelectric point (pI), predicted subcellular location, and GenBank accession number. Table S1 shows the conserved PAP motifs. The relative molecular masses of 31 proteins were estimated to be 27.91 to 71.10 kDa, and the length of the MdPAP proteins were approximately 252 to 656 aa.

Bioinformatics was used to predict the subcellular locations of the PAP proteins. MdPAP1, MdPAP2, MdPAP13, MdPAP14, MdPAP22, MdPAP28, and MdPAP31 are located in the nucleus. The MdPAP6 protein is localized mainly in the chloroplast and nucleus. MdPAP12 is located in the cytoplasm and nucleus. MdPAP7 and MdPAP19 are extracellular. The other proteins are expressed in the cell wall and nucleus (Table 1).

For PAPs, we compared multiple sequences from eukaryotes and prokaryotes and found that seven invariant residues in five conserved amino acid sequence regions ([DxG/GDxxY/GNH(D,E)/VxxH/GHxH]) were the ones required for metal coordination [40]. By conservative domain analysis, 31 MdPAP sequences were found. The carboxyl terminus of the column has a conserved structure composed of five conserved motifs, and only MdPAP31 lacks a motif (Table S1). In MdPAP1, 2, and 6, the “Y” residue is substituted with an “F” in the second block (GDXXY). In contrast, in MdPAP17, the “H” residue is replaced by an “I” in the fifth block (GHXH). Owing to its high homology with PAP, it is still classified as an MdPAP (Table S1).

**Figure 1:**
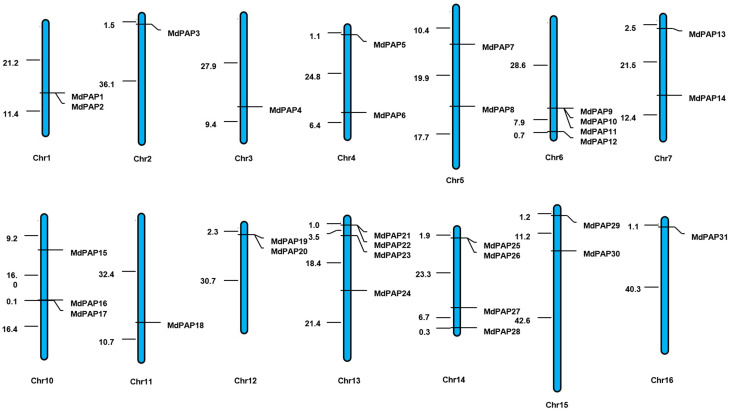
Illustrates the locations of purple acid phosphatases (*PAP*) gene family members on the apple chromosomes.

In all, 31 *MdPAP* genes are distributed on 14 apple chromosomes (Figure 1). Chromosomes 2, 3, 11, and 16 each contain one gene; chr 1, 4, 5, 7, 12, and 15 have two genes; chr 10 have three each; and chr 6, 13, and 14 have four genes.

### 2.2. Molecular Cloning of MdPAP Genes from Apple

The 11 *MdPAP* genes that cloned were submitted to the NCBI database (Table 1). Upon comparing these sequences with previously published apple gene predictions [41], we identified four anomalies: redundant fragments and deletions. This redundancy was identified within the central segment of MD03G1203900. Compared with the cloned sequences from *M. hupehensis*, the predicted MD10G1067000 sequence is deficient in a sequence region. However, the cloned sequences demonstrate a high degree of similarity to the predicted coding sequences within the apple genome, irrespective of whether they represent mispredicted, redundant, or deleted fragments.

### 2.3. Phylogenetic Analysis of the PAP Gene in Plants

**Figure 2:**
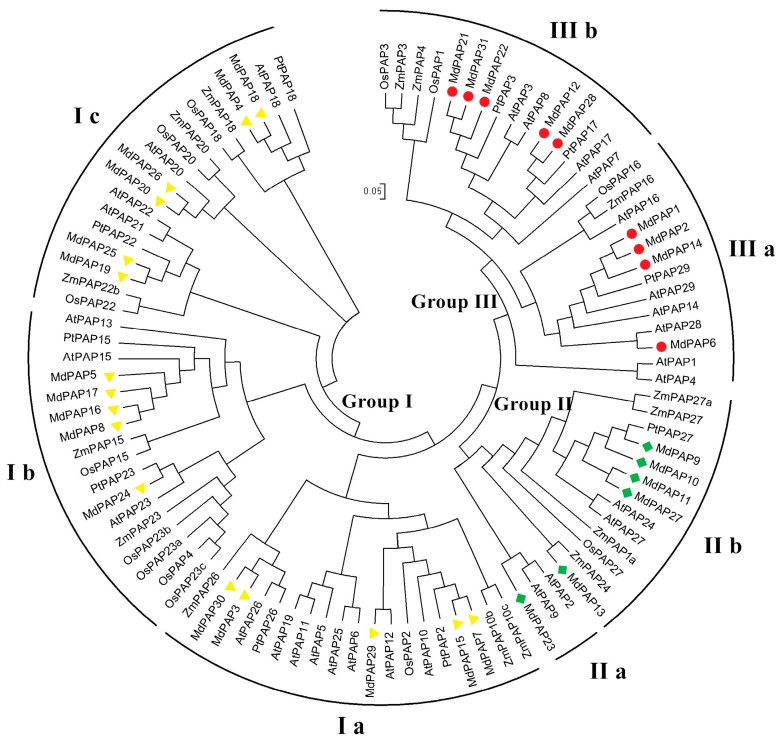
Phylogenetic analyses of purple acid phosphatases (PAP) genes in *Arabidopsis thaliana* (At), *Oryza sativa* (Os), *Populus trichocarpa* (Ptr), and *Malus domestica* (Md). Using MEGA6.0 program, the rootless phylogenetic tree was constructed by NJ method. The phylogenetic tree is divided into three groups and seven subgroups, each marked with different colors.

To elucidate the evolutionary and phylogenetic relationships among PAPs in apple, as well as other plant species, we conducted a thorough analysis of their full-length protein sequences. The phylogenetic trees generated from this analysis revealed that plant PAP proteins could be categorized into three distinct clusters (Groups I, II, and III) and seven subclusters (Groups Ia, Ib, Ic, IIa, IIb, IIIa, and IIIb) using the NJ method (Figure 2). The three clusters corresponded to the clustering of PAP families in *Arabidopsis* [7]. The largest cluster I contained 19 MdPAP members and three subclusters, followed by Cluster II, which contained seventeen members and two subclusters; Cluster III had six members and two subclusters. Phylogenetic analysis revealed that the *MdPAPs* presented a closer evolutionary relationship with the dicot species *Arabidopsis* and *Populus* than with the monocot species rice and maize. Two sets of paralogous genes were also found in apple: *MdPAP3/MdPPAP30* and *MdPAP4/MdPPAP18.*

### 2.4. Analysis of MdPAP Structure and Motifs in Apple

**Figure 3:**
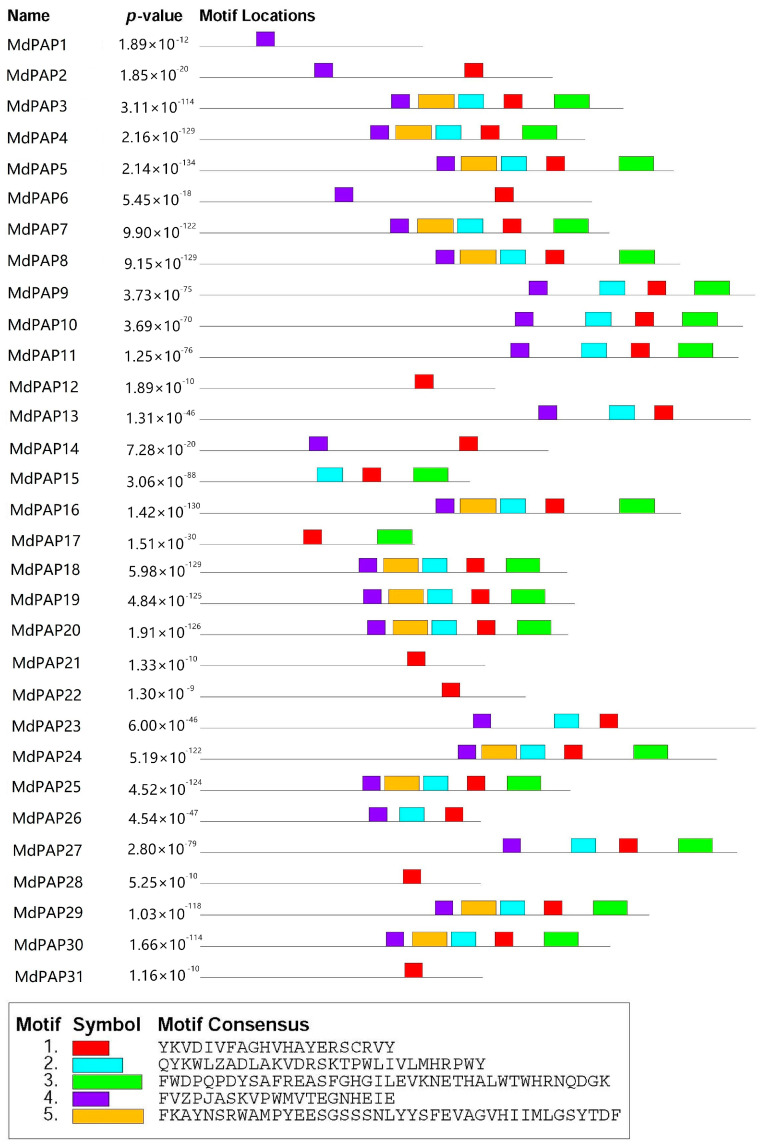
The architecture of the conserved domain of apple purple acid phosphatases (PAP) proteins. The abundance of amino acids in each motif of PAP proteins was shown in the sequence.

The protein sequence alignments were obtained via MEME software (https://meme-suite.org/meme/tools/meme, accessed on 2 February 2024). The motif analysis revealed the presence of five conserved motifs (1–5) within the acquired PAP family members. The results revealed that MdPAP3–5, MdPAP16, MdPAP18–20, MdPAP24, MdPAP25, MdPAP29, and MdPAP30 contained one–five motifs. The amino acid compositions of all the motifs are shown in Figure 3.

**Figure 4:**
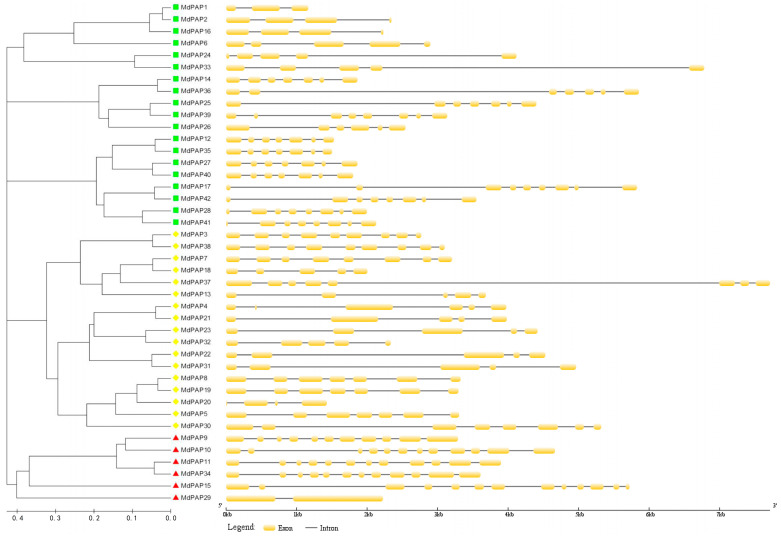
A schematic diagram of exon/intron structures for purple acid phosphatases (*PAP*) genes in apple; yellow boxes represent exons while gray lines signify introns. The yellow diamonds represent Groups I, the red triangles represent Groups II, and the green squares represent Groups III.

We conducted a comparative analysis between the full-length cDNA sequence and the genomic DNA sequence corresponding to each *MdPAP* gene was analyzed to determine the number and location of exons and introns. This analysis was performed on a total of 31 coding sequences, and each coding sequence was disrupted by 3 to 13 introns (Figure 4).

### 2.5. Cis-Acting Element Analysis in MdPAP Promoter Regions

**Figure 5:**
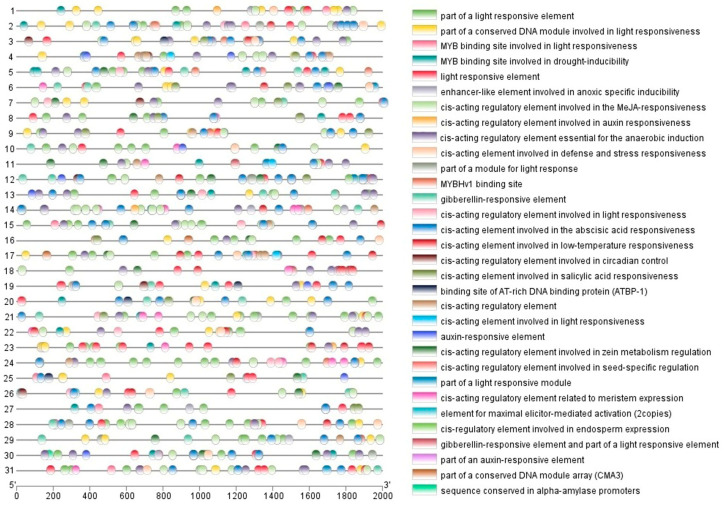
The cis-acting elements within the promoters of purple acid phosphatases (*PAP*) genes in the apple genome. Different color boxes represent the different identified cis-acting elements.

The region of the *MdPAP* gene promoter region of 2000 bp upstream was downloaded and analyzed via the PlantCARE database to understand the presence of cis-acting elements. A series of cis-regulatory elements, including MYB binding sites, MeJA responsive elements, auxin responsive elements, defense and stress responsiveness, and salicylic acid responsiveness, were found in the upstream regions of the *MdPAP* genes (Figure 5).

### 2.6. Expression Profiles of PAP Genes in Different Apple Tissues

**Figure 6:**
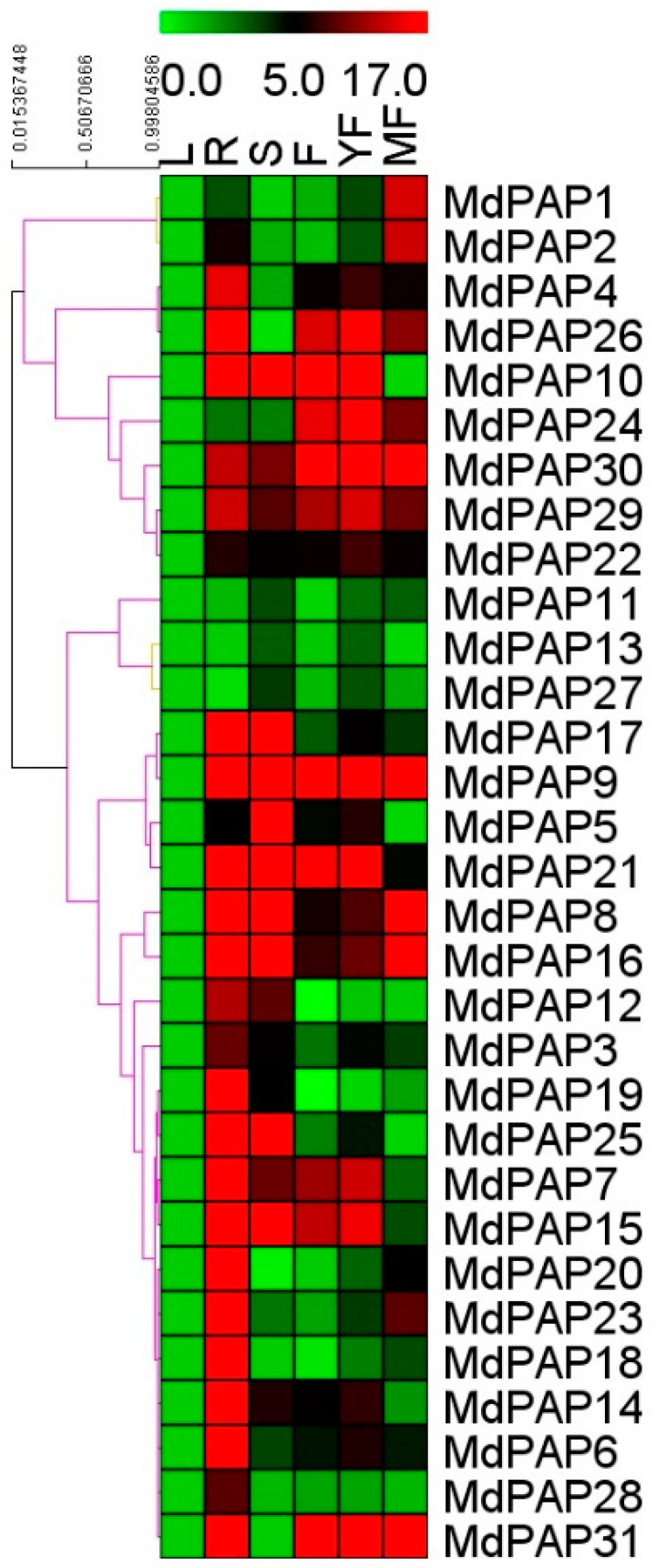
Quantitative real-time PCR analysis of selected apple purple acid phosphatases (*PAP*) genes expressed in leaves (L), roots (R), stems (S), flowers (F), young fruits (YF), and mature fruits (MF) from *Malus hupehensis*.

qRT-PCR analysis revealed that all 31 *MdPAP*s were ubiquitously expressed in the roots, leaves, stems, flowers, and young and mature fruits of apple (Figure 6 and Figure S1). All the *MdPAPs* were expressed in the leaves. In the roots, almost all the *MdPAP* genes were strongly expressed, except for *MdPAP10*, *MdPAP12*, and *MdPAP27*. The expression of *MdPAP4*, *MdPAP*6–10, *MdPAP*12, *MdPAP*15–21, *MdPAP*23, *MdPAP*25, *MdPAP*26, and *MdPAP*28–31 was obviously greater in the roots than in the leaves. In stem tissue, half of the *MdPAPs* presented relatively high expression. Eight genes—*MdPAP1*, *2*, *4*, *18*, *20*, *26*, *28*, and *31—*had relatively low expression in the stems, whereas *MdPAP5*, *7–10*, *15–17*, *21*, and *25* were most strongly expressed in the stems of apple plants. The transcripts of *MdPAP7–9*, *15*, *21*, *24*, *25*, and *29–31* were highly expressed in the flowers, whereas the other genes presented lower expression in the flowers. In young apple fruit tissue, the transcript levels of only five *MdPAPs* were relatively low, and *MdPAP7–10*, *15*, *16*, *21*, *24*, *26*, and *29–31* were highly expressed in young fruits. *MdPAP1*, *2*, *8*, *9*, *16*, *23*, *24*, *26*, and *29–31* were strongly expressed in the mature fruits, whereas the other genes were expressed at lower levels in the mature fruits.

### 2.7. Effects of Various Stresses on MdPAP Expression

**Figure 7:**
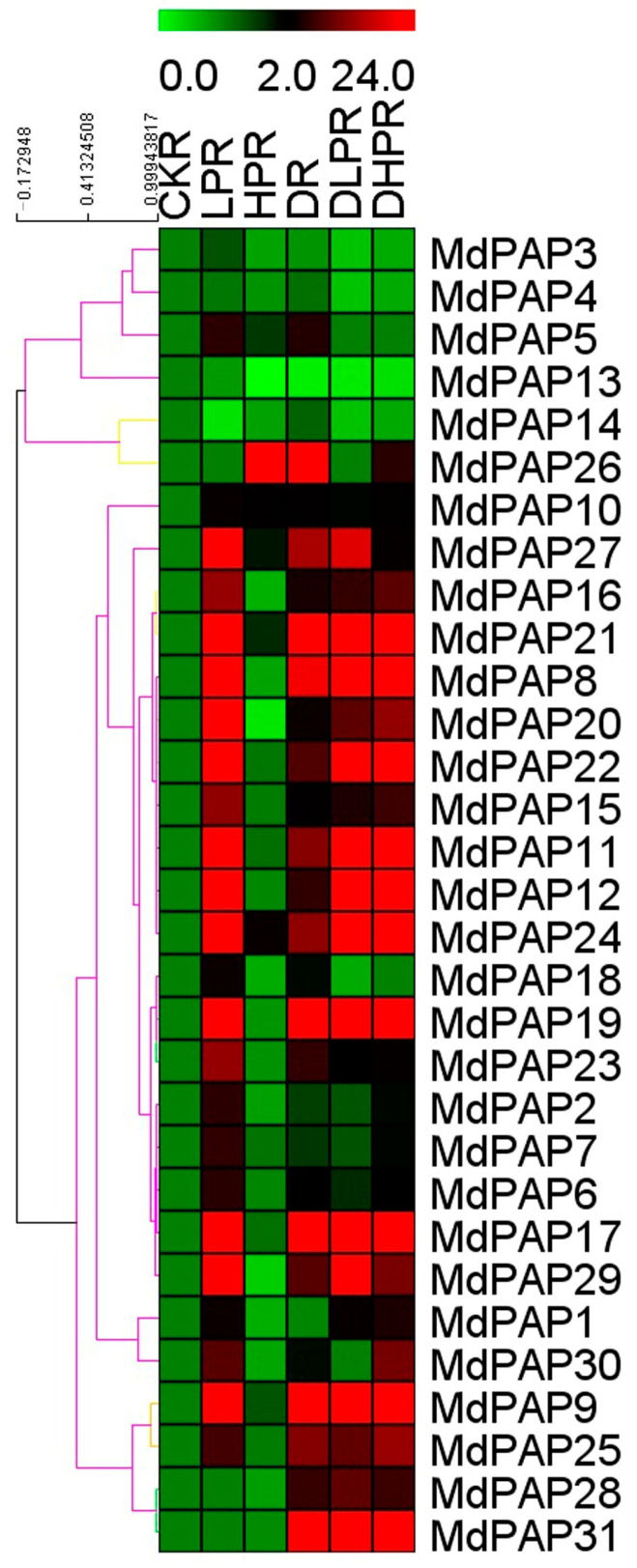
The expression profiles of purple acid phosphatases (*PAP*) genes in the roots of *Malus hupehensis*. Heatmap indicates the expression of *PAP* genes across after 15 d of phosphorus stress; samples were taken from roots exposed to low-P condition (LLP), high-P condition (LHP), drought condition (LD), drought with low-P condition (LDLP), or drought with high-P condition (LDHP). All transcript levels were normalized to the corresponding levels in the non-stressed control (LCK).

After 15 d of exposure to low-P stress, the majority of the *MdPAP* genes were upregulated in the roots (Figure 7 and Figure S2). In particular, the genes encoding *MdPAP7*, 8, 10, 11, 14–16, 19–23, 27, and 28 were highly expressed under low-P conditions, and only the *MdPAP12* and *MdPAP13* genes were downregulated, suggesting that most *MdPAP* genes may play a role in the response to P starvation. Under high-P stress, the expression of all genes remained largely unchanged by this treatment, with only the *MdPAP26* gene being upregulation. Under drought conditions, the expression of *MdPAP8*, *MdPAP9*, *MdPAP11*, *MdPAP17*, *MdPAP19*, *MdPAP20–22*, and *MdPAP24–31* in roots was increased significantly compared with that in the control, and the expression of only *MdPAP12* was increased. In response to the synergistic effects of low-Pi and drought conditions, the expression of *MdPAP8*, *MdPPAP9*, *MdPAP11*, *MdPAP17*, *MdPAP19–21*, *MdPAP24*, *MdPAP25*, *MdPAP27–29*, and *MdPAP31* significantly increased in the roots, whereas the expression of five other genes—*MdPAP3*, *MdPAP4*, *MdPAP13*, *MdPAP14*, and *MdPAP18—*indicated the decreasing expression in roots. Under high phosphorus and drought stresses, 14 of *31 MdPAP* genes were obviously up-regulated, only the expression of *MdPAP3–5*, *MdPAP13*, *MdPAP14*, and *MdPAP18* were marginally reduced when plants were exposed to the combined treatment, while other genes remained stable.

**Figure 8:**
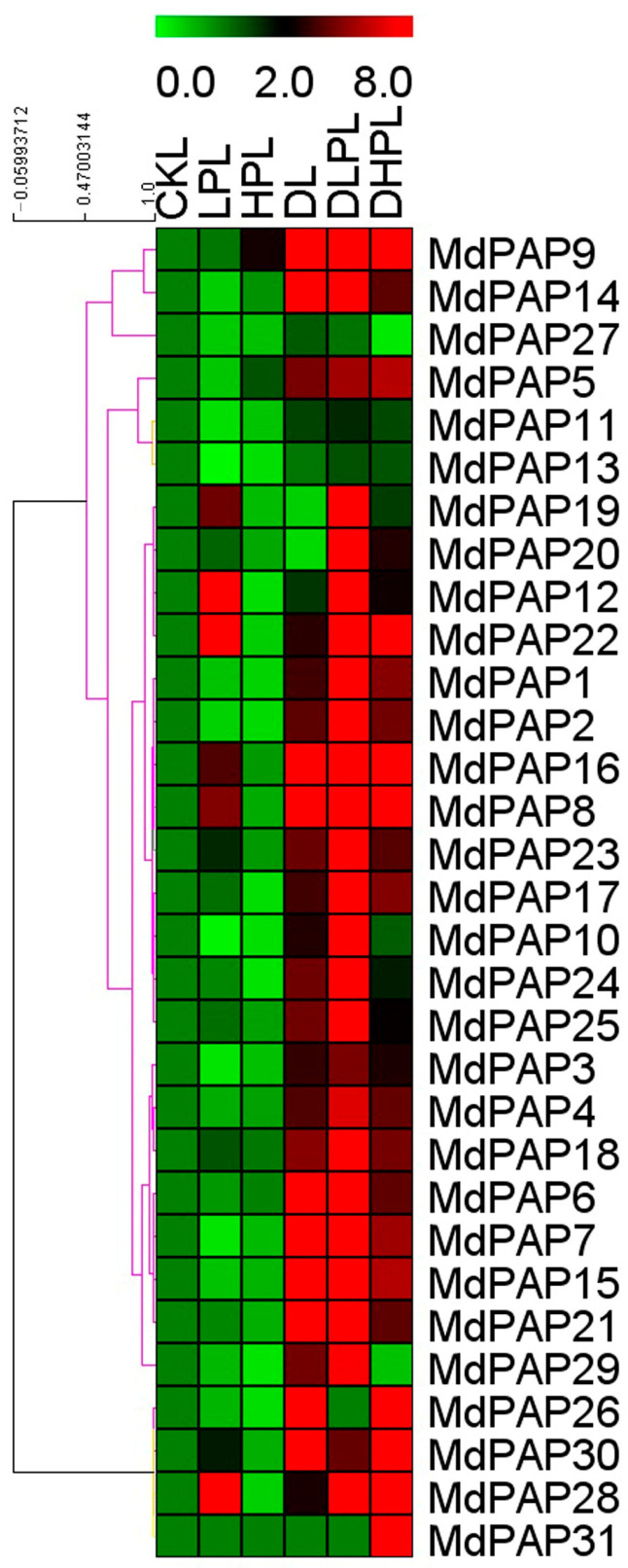
The expression of purple acid phosphatases (*PAP*) genes in leaves of *Malus hupehensis*. Heatmap indicates *PAP* genes expression across after 15 d of phosphorus condition; samples were taken from leaves exposed to low-P condition (LLP), high-P condition (LHP), drought condition (LD), drought with low-P condition (LDLP), or drought with high-P condition (LDHP). All transcript levels were normalized to the respective corresponding levels in the non-stressed control (LCK).

In apple leaves, P-starvation stress caused the *MdPAP8*, *MdPAP12*, *MdPAP16*, *MdPAP19*, *MdPAP22*, *MdPAP23*, *MdPAP28*, and *MdPAP30* genes to be highly expressed, whereas the other genes were unchanged or downregulated (Figure 8 and Figure S3). With the addition of excessive P, the expression of 9 *MdPAP* genes slightly increased compared with that in the control. However, the expression levels of the other genes either decreased or remained stable after 15 d of high-P treatment. In the context of drought conditions, 24 *MdPAP* genes were significantly upregulated. Only the *MdPAP19* and *MdPAP20* genes were downregulated, and the expression of *MdPAP11–13*, *MdPAP27*, and *MdPAP31* did not change under drought stress in the leaves. The expression of *MdPAP13*, *MdPAP26*, *MdPAP27*, and *MdPAP31* remained stable under combined starvation and drought stress. All the *MdPAP* genes were similarly induced by these stresses. When drought and high-P stresses were concurrently applied, only the expression of *MdPAP27* and *MdPAP29* decreased. The expression of *MdPAP3*, *MdPAP10–13*, *MdPAP19*, *MdPAP20*, *MdPAP24*, and *MdPAP25* remained stable, whereas that of most other genes was upregulated.

## 3. Discussion

Phosphorus, a vital nutrient, profoundly impacts plant growth and metabolism [2]. Therefore, the relationship between the PAP protein and P utilization can reflect the adaptation of plants to different environments. This study concentrated on apple purple acid phosphatases and conducted a comprehensive genome-wide investigation and characterization of these genes. The aim was to identify potential candidate PAPs for future functional analyses.

### 3.1. The PAP Genes in Apple

In the apple genome, 31 *MdPAP* genes were identified and compared with the PAP sequences of rice and *Arabidopsis*. The number identified is slightly greater than that previously reported in rice and *Arabidopsis* [7], tomato [12,13,42], and coffee genomes [43], and was comparable to that reported in soybean and maize genomes [16,17].

Phylogenetic analysis revealed that the MdPAPs can be classified into three groups and seven subgroups in apple, which aligns with previous research conducted on *Arabidopsis*, rice, soybean, and maize [7,15,16,17]. Groups I and II of the *Arabidopsis* PAP family are distinguished by their high-molecular-weight PAPs, while Group III is composed of low-molecular-weight mammalian-like PAPs [7]. In the phylogenetic analysis of PAPs in maize, *Arabidopsis*, rice, and apple, these three groups were all observed (Figure 3).

The proteins of MdPAP have 255 to 656 amino acids, similar in size to AtPAP proteins in *Arabidopsis*, rice and maize, which contain 242 aa to 656 aa [5], 335 aa to 1025 aa [15], and 235 aa to 633 aa, respectively [17]. Since most of these MdPAPs (Table 1) are similar in length to those members in rice, maize, and *Arabidopsis*, we suggest that proteins in this family are highly conserved across species. The fifth of the 31 PAPs in apple, Group I, aligns with the 15 members of the *Arabidopsis* group and exceeds the 13 members of the maize group. Notably, subgroup Ia, which includes AtPAP5, AtPAP6, AtPAP11, AtPAP19, and AtPAP25, was identified as being deleted in the apple genome. This deletion is also present in both maize and rice [15,17]. There were six Group II apple PAPs, fewer than five *Arabidopsis* PAPs, and fewer than eleven maize in Group II. Group III was similar in size in apple, *Arabidopsis*, and maize (nine, nine, and eleven members, respectively).

Sequence analysis revealed that 30 apple PAPs contained the amino acid sequences of all five blocks and seven invariant residues, which are integral to linking the bimetallic nuclear center found in known PAPs (Table 1). The residual PAP protein (MdPAP31) is devoid of the first block, XDXG. Despite this, the gene was still classified as a putative PAP gene because its overall amino acid sequence demonstrated significant homology with recognized PAPs. However, for four out of the thirty-one MdPAPs, this classification was not applicable (Table 1), and their potential metal-linked residues differ from those of typical PAPs. In MdPAP1, MdPAP2, and MdPAP6, the potential metal-associated residue species generally exhibit greater similarity to those identified in AtPAP14 and AtPAP16 [7]. In CaPAP16, 28 and 29, the “Y” residue is replaced by an “F” in the second block (GDXXY) [18]. In MdPAP17, the deletion of one out of seven invariant residues is due to a slight change in the amino acid composition of one out of five sequence elements of the amino acid composition, and the amino acid sequence comparison does not infer that these amino acids are metal-linked residue reserved positions (Table S1 and Figure 3). AtPAP13 lacks four of seven invariant residues and has also been found to possess exonuclease or phosphodiesterase activity and is still treated as a PAP protein [7]; CaPAP18a and CaPAP23a lack a fifth block (GHXH) [18]. However, their sequence still have significant homology with known PAPs. Therefore, these proteins are considered putative PAPs. Similarly, MdPAP1, MdPAP2, MdPAP6, and MdPAP17 are regarded as potential PAPs because their overall amino acid sequences are highly homologous to those of to known PAPs.

Among the 31 coding sequences for the MdPAP proteins, each is disrupted by two to eleven introns (Figure 4). Compared with the gene structures of PAPs from rice and *Arabidopsis* [7,15], those of *MdPAPs* are nearly identical in the number of exons to those of the other two species. These findings suggest that the gene structure in apple is similar to that in *Arabidopsis* and rice. There are two sets of paralogous genes in *MdPAPs*: *MdPAP3/MdPAP30* and *MdPAP4/MdPAP18* (Figure 4).

We identified a series of cis-regulatory hormone/stress-related elements in the upstream regions of these genes, such as MYBs and TATA-boxes, suggesting that MdPAPs may play a key role in resistance to abiotic stresses and responses to hormonal stresses (Figure 5).

### 3.2. Expression Profiles of MdPAPs in Different Apple Tissues

The majority of *AtPAPs* and *ZmPAPs* are expressed predominantly in roots, yet their presence is also discernible in leaves and flowers [17,44]. We also detected *MdPAP* transcripts in leaves, roots, stems, flowers, young fruits, and mature fruits (Figure 6). All the *MdPAPs* are expressed in the leaves. The expression of most *MdPAPs* is strong in the roots, especially that of *MdPAP11*, *MdPAP13*, and *MdPAP27*. The three genes under discussion are classified within Group II, mirroring the type II pattern observed in *Arabidopsis*. Transcripts from this Group II pattern were identified in stem, leaf, flower, and silique tissues; however, they were undetectable in root tissues [44]. This may be because the transcription of Group II members of AtPAP and MdPAP predominantly occurs in reproductive organs.

In apple, *MdPAP5*, *MdPAP7–MdPAP10*, *MdPAP12*, *MdPAP15-MdPAP17*, *MdPAP21*, *MdPAP25*, *MdPAP29*, and *MdPAP30* are expressed predominantly in the stems, whereas *MdPAP19*, *MdPAP26*, and *MdPAP31* are expressed at lower levels in the stems. The expression of *CaPAPs* has been observed in various tissues, including the roots, shoots, mature leaves, flower buds, and young pods. Among these genes, the lowest expression of *CaPAPs* was noted in the shoot tissue [18].

*MdPAP7*, *MdPAP9*, *MdPAP15*, *MdPAP21*, *MdPAP24*, *MdPAP26*, *MdPAP29–31* are highly expressed in apple flowers, and the expression of these genes is basically the same as that in young fruits, and the overall trend is upregulation. All 28 *AtPAP* members were transcribed in flower tissues, and the relative expression levels of *AtPAP6*, *11*, *14*, *19*, *23*, *24*, and *25* were the highest in flower tissues [44].

In the mature fruits, *MdPAP1*, *MdPAP2*, *MdPAP8*, *MdPAP9*, *MdPAP16*, *MdPAP23*, *MdPAP24*, *MdPAP26*, and *MdPAP29–31* are highly expressed. *AtPAP20* is uniquely expressed in *Arabidopsis* siliques and flowers [44], and the rice homologs of *AtPAP20* and *OsPAP20a* may also be expressed in reproductive organs [15]. During the ripening of the apple fruits, the expression levels of *MdPAP1*, *MdPAP2*, *MdPAP9*, *MdPAP16*, *MdPAP30*, and *MdPAP31* are continuously upregulated, indicating that these genes may be involved in the development of apple ripening. Several *Arabidopsis PAPs* are also predominantly expressed in flowers and siliques; for example, the *AtPAP23* gene has a dynamic expression pattern during flower development [44]. In the reproductive tissues of maize, many PAP transcripts also strongly accumulate in tassels [17], suggesting their potential function in flower development [44]. *CaPAPs* exhibit robust expression in flower buds and young pods, suggesting that they play pivotal roles during reproduction [18].

### 3.3. Effects of Different Phosphorus Concentrations and Drought Conditions on PAP Gene Expression in Apple

In higher plants, since the expression of multiple *PAPs* is significantly induced under phosphate starvation stress, it is speculated that PAPs play a crucial role in phosphate metabolism. At lower P stress, 11 *ZmPAPs* in leaf transcripts accumulate to significantly greater levels. All the *ZmPAPs* in the roots are expressed at higher levels under LP conditions [17]. The expression of 10 *PAP* genes is elicited by LP stress in rice [15]. The majority of PAP family genes expressed in *Arabidopsis* roots are upregulated in plants deprived of phosphate [45]. In the roots of wheat under LP stress, 57 *TaPAPs* are highly expressed [21]. We also observed that P starvation induced increased expression: 24 *MdPAPs* were induced in roots, and the expression of *MdPAP8*, *MdPAP11*, *MdPAP12*, *MdPAP17*, *MdPAP19*, *MdPAP21*, *MdPAP24*, and *MdPAP29* increased greatly compared with that of the control (Figure 7). In this study, we found that P starvation induced the expression of 24 apple *PAP* genes, suggesting their significant role in apple acclimation to low-P stress.

While most *MdPAP* genes are upregulated in the roots, six of them—*MdPAP8*, *MdPAP12*, *MdPAP16*, *MdPAP19*, *MdPAP22*, and *MdPAP28—*are also induced in the leaves, whereas the expression of the other genes either remains stable or decreases in these tissues. As a result, genes expressed in roots may demonstrate increased sensitivity to the limited phosphorus supply in the soil. An elevated accumulation of *ZmPAP30a* transcripts was noted in both shoots and roots under phosphorus deficiency [17]. The expression of *OsPAP1d*, *OsPAP10c*, *OsPAP23*, and *OsPAP27a* was significantly elevated following phosphorus deficiency [15]. *StPAP3* and *StPAP7* were upregulated in potato roots under low-P treatment [29]. Under LP conditions, the expression of *MdPAP8* and *MdPAP12* significantly increased (Figure 7 and Figure 8); therefore, these two genes are good candidates for low-P-responsive markers.

When plants are exposed to excess P, *MdPAP10* and *MdPAP26* are upregulated in the roots, and *MdPAP5* and *MdPAP9* are upregulated in the leaves. These genes are located in the cell wall and nucleus, suggesting that their proteins regulate the absorption and distribution of phosphorus in plants in high-phosphorus environments.

PAP also contributes to plant resistance and responses to abiotic stress [46]. The overexpression of *AtPAP15* increased tolerance to salt and osmotic stresses [47]. A pronounced downregulation of *StPAP3* was observed in the leaves under drought stress [29]. Under drought stress, the expression of *MdPAP8*, *MdPAP9*, *MdPAP17*, *MdPAP19*, *MdPAP21*, *MdPAP24–27*, and *MdPAP31* is significantly upregulated in roots subjected to stress, whereas the expression of *MdPAP8* and *MdPAP9* is notably increased in leaves. These findings indicate that these genes are upregulated under drought conditions.

Plants in their natural environment do not only face a single stress but also may encounter multiple stresses [48]. The combination of multiple stresses produces a unique pattern of gene expression, which is distinct from the study of either stress individually [49]. Under low-P stress, the expression of *MdPAP12* and *MdPAP22* is highly responsive, either independently or in conjunction with drought stress. However, when drought stress is the sole inducer, the expression of these genes remains consistent. The upregulation of *MdPAP12* and *MdPAP22* appears to be induced solely by phosphorus starvation, implying that these two proteins may play a role in the low phosphorus tolerance observed in apple leaves. Regardless of P status, the expression of *MdPAP31* in roots, and that of *MdPAP3*, *MdPAP4*, *MdPAP6*, *MdPAP7*, *MdPAP15*, *MdPAP18*, *MdPAP21*, *MdPAP24*, and *MdPAP25* in leaves were upregulated under drought conditions, indicating that these genes are not modulated by the level of P. Nevertheless, the alterations in the expression of the *MdPAP12*, *MdPAP22*, and *MdPAP24* genes in response to drought are predominantly influenced by the phosphate concentration within the root system. Consequently, these findings imply that the uptake of P by plants under drought conditions may be associated with alterations in *PAP* expression. This could serve as an indicator of the degree of drought tolerance exhibited by these different stressed plants.

The expression of 27 *MdPAP* genes is upregulated in apple leaves, and the expression of 20 genes is induced in roots under drought with low-phosphorus conditions, indicating that the above-ground parts of plants are more affected than the underground parts. Consequently, it is imperative for the above-ground parts of plants not only to resist drought but also to enhance the reuse of phosphorus. Compared with single stress, the *MdPAP* gene presented a positive response under double stresses, and the underlying mechanism of the regulation of *MdPAP* genes needs further study.

## 4. Materials and Methods

### 4.1. Plant Materials and Stress Treatments

*Malus hupehensis* var. *pingyiensis*, which originated in China, and has high genetic stability and tolerance to waterlogged conditions, was used as the plant material [50]. Samples of the apple roots, leaves, shoot tips, stems, flowers (collected 5 days after blooming, DAB), young fruits (15 DAB), and mature fruits (122 DAB) were procured from 7-year-old *M. hupenensis* trees.

For hydroponics experiments, seeds of *M. hupenensis* were subjected to a stratification process in sand at a temperature of 4 ^◦^C for 40 days. These germinating plants were subsequently cultivated in plastic pots, each measuring 12 cm × 12 cm, filled with sand for a period of 60 d. The detailed procedures are described in our previous research [51]. The seedlings were randomly allocated into 6 distinct groups for a duration of 15 d. The control group (CK) was supplied with a standard 1/2-strength Hoagland nutrient solution enriched with 500 μM KH_2_PO_4_. The low P stress (LP) was placed in 1/2-strength Hoagland nutrient containing 5 μM KH_2_PO_4_. The high P stress (HP) solution was placed in 1/2-strength Hoagland nutrient solution with 5 mM KH_2_PO_4_. The drought stress (D) was set at −0.75 MPa. The drought stress with low-P condition (DLP) combined 5 μM KH_2_PO_4_ and −0.75 MPa. The drought stress and high-P condition (DHP) combined 5 mM KH_2_PO_4_ and −0.75 MPa. Each treatment was performed in triplicate with 30 plants per replicate (Table 2). The roots and leaves of the seedlings were distinctly collected, rapidly frozen in liquid nitrogen, and subsequently preserved at a temperature of −80 °C for the purpose of expression analysis.

### 4.2. Identification of the PAP Gene Family and Chromosome Locations of PAP in Apple

The established PAP amino acid sequences from *Arabidopsis thaliana*, *Populus trichocarpa*, and *Oryza sativa* were employed as search queries against the apple genome database (https://iris.angers.inra.fr/gddh13/, accessed on 2 January 2024) [41]. The conserved structure of the protein was determined via the HMMER (http://www.cbs.dtu.dk/services/TMHMM-2.0/, accessed on 8 January 2024) and SMART (http://smart.embl-heidelberg.de/, accessed on 8 January 2024) tools [52]. The Genome Database for Rosaceae (http://www.rosaceae.org/, accessed on 4 January 2024) was sourced from locational data pertaining to *PAP* genes [53]. The molecular weights of the *MdPAP* genes, in conjunction with their physical and chemical attributes, were scrutinized utilizing tools available on the ExPASy online website (http://www.expasy.org/, accessed on 12 January 2024) [54]. The subcellular localization of MdPAP was ascertained utilizing WoLF PSORT (http://www.genscript.com/wolf-psort.html, accessed on 14 January 2024) [55]. The chromosomes were mapped by MapGene2Chrom (http://mg2c.iask.in/mg2c_v2.1/, accessed on 4 January 2024) [56].

### 4.3. Phylogenetic, Exon/Intron Organization and Motif Analysis of the MdPAP Genes

The phylogenetic trees for the PAP protein sequences of apple, *Arabidopsis*, rice, maize, and poplar were constructed with neighbor-joining using MEGA 6.0 software. The exon/intron organization was depicted using the Gene Structure Display Server (http://gsds.cbi.pku.edu.cn/, accessed on 18 January 2024) [57]. Motif analysis of PAPs was conducted using MEME (https://meme-suite.org/meme/tools/meme, accessed on 2 February 2024) with 5 putative conserved motifs [58].

### 4.4. RNA Extraction, qRT-PCR Analysis, and Molecular Cloning of PAP Genes in Apple

Total RNA was isolated from apple tissues utilizing the CTAB protocol [59]. The concentration of RNA was assessed spectrophotometrically and confirmed by ethidium bromide staining of a 1.2% (*w*/*v*) agarose gel. The RNA was reverse transcribed into cDNA using a MightyScript Plus first-strand cDNA synthesis kit (gDNA digester) (Sangon Biotech, Shanghai, China). qRT-PCR was performed with the SYBR Prime Script RT-PCR Kit II (TaKaRa, Osaka, Japan). The relative expression level of each gene was ascertained through the application of the 2^−ΔΔCt^ method [60].

The *MdPAP* sequences were amplified using leaf cDNA as the template. Specific primers (Table S2) for gene cloning were designed based on the revised putative sequences. Polymerase chain reactions (PCRs) were performed with PrimeSTAR^®^ HS DNA Polymerase (TaKaRa, Dalian, China), and amplification conditions were empirically optimized. The PCR products were added to the 3′-termini using TaqDNA Polymerase (Fermentas, Waltham, MA, USA) and cloned into the pMD19-T vector (TaKaRa, Dalian, China). Afterward, positive clones were sequenced. Gene nomenclature was assigned based on chromosome orders.

## 5. Conclusions

In summary, a total of 31 *MdPAP* genes were identified, characterized into three clusters and distributed on 14 chromosomes. After analyzing the phylogeny, unique exon/intron structures, conserved motif distributions, and the cis-acting elements of the *MdPAP* genes, qRT-PCR revealed that the expression of the *MdPAP* genes is stimulated by both phosphate deprivation and excess, in addition to drought stress. Our work contributes to the enhanced comprehension of the evolution and subsequent analysis of the activities and functions associated with the *PAP* gene family. Although the functions of these *MdPAPs* remain to be elucidated, our data suggest that they are involved in plant adaptation to stress. If these factors can be exploited to improve apple tolerance to low-P stress, our study will also provide a basis for future functional studies of the PAP family in apple.

## Figures and Tables

**Table 1:** Properties of purple acid phosphatases (PAP) identified from the *Malus domestica* genome.

Gene	Locus	Genome Position	ORF Length (bp)	Amino Acid	Number of Exons	MW (kDa)	pI	Subcellular Location Prediction	GenBank Accession Number
*MdPAP1*	MD01G1097200	Chr01:21160964-21161200	768	255	3	27.91	4.71	Nucleus.	
*MdPAP2*	MD01G1098300	Chr01:21210868-21211737	1224	407	4	44.31	7.70	Nucleus.	MT465934
*MdPAP3*	MD02G1021200	Chr02:1496528-1500542	1461	486	10	55.79	7.32	Cell wall. Nucleus.	MT465935
	MD02G1021400	Chr02:1501609-1502368	171	56					
*MdPAP4*	MD03G1203900	Chr03:27852127-27856406	1305	434	5	48.80	6.17	Cell wall. Nucleus.	MT465936
	MD03G1203400	Chr03:27804640-27805369	423	140					
*MdPAP5*	MD04G1009700	Chr04:1101652-1104956	1635	544	7	61.23	5.32	Cell wall. Nucleus.	
	MD04G1009800	Chr04:1105736-1109257	1980	659					
*MdPAP6*	MD04G1168800	Chr04:25924849-25928095	1287	428	5	50.57	5.98	Chloroplast. Nucleus.	
*MdPAP7*	MD05G1059300	Chr05:10350920-10354674	1329	442	8	53.71	6.63	Extracell.	
*MdPAP8*	MD05G1174000	Chr05:30226979-30230752	1656	551	7	62.26	5.03	Cell wall. Nucleus.	
*MdPAP9*	MD06G1142200	Chr06:28584454-28587742	1914	637	11	70.98	6.09	Cell wall. Nucleus.	
*MdPAP10*	MD06G1142300	Chr06:28606916-28612194	1872	623	12	69.97	5.95	Cell wall. Nucleus.	
*MdPAP11*	MD06G1142400	Chr06:28612560-28616918:	1857	618	12	69.29	5.67	Cell wall. Nucleus.	MT465937
*MdPAP12*	MD06G1233500	Chr06:36463094-36465228	1020	339	7	38.50	6.11	Cytoplasm. Nucleus.	
	MD06G1233400	Chr06:28606916-28612194	618	205					
*MdPAP13*	MD07G1029700	Chr07:2479289-2485298	1899	632	13	70.12	6.62	Nucleus.	
	MD07G1028100	Chr07:2348608-2349147	270	89					
	MD07G1028400	Chr07:2362088-2365556	1275	424					
	MD07G1028500	Chr07:2369779-2370474	216	71					
*MdPAP14*	MD07G1164800	Chr07:23966820-23969514	1161	386	4	41.94	6.00	Nucleus.	MT465938
*MdPAP15*	MD10G1067000	Chr10:9216263-9219031	1026	341	5	39.84	6.39	Extracell.	MT465939
*MdPAP16*	MD10G1161900	Chr10:25263414-25266989	1659	552	7	62.44	5.29	Cell wall. Nucleus.	MT465940
*MdPAP17*	MD10G1162700	Chr10:25335832-25337261	744	247	4	27.69	6.18	Cell membrane. Nucleus.	
*MdPAP18*	MD11G1220800	Chr11:32387226-32391632	1305	434	5	49.10	6.18	Cell wall. Nucleus.	MT465940
*MdPAP19*	MD12G1023300	Chr12:2334733-2339497	1332	443	5	50.08	6.02	Cell wall. Nucleus.	
*MdPAP20*	MD12G1023400	Chr12:2356138-2360751	1308	435	5	48.39	5.60	Cell wall. Nucleus.	MT465941
*MdPAP21*	MD13G1016000	Chr13:1023442-1028285	1014	337	7	37.86	4.94	Cell wall. Nucleus.	
*MdPAP22*	MD13G1016100	Chr13:1028992-1031649	930	309	6	43.42	6.23	Nucleus.	
*MdPAP23*	MD13G1064900	Chr13:4484193-4486861	1974	657	2	73.21	5.61	Cell wall. Nucleus.	MT465942
*MdPAP24*	MD13G1231100	Chr13:22917107-22922719	1836	611	8	68.30	5.64	Cell wall. Nucleus.	
*MdPAP25*	MD14G1020300	Chr14:1907612-1912812	1317	438	5	49.51	5.75	Cell wall. Nucleus.	
*MdPAP26*	MD14G1020400	Chr14:1915448-1917990	1134	377	5	36.42	6.09	Cell wall. Nucleus.	
*MdPAP27*	MD14G1157600	Chr14:25180181-25183789	1905	634	12	71.03	5.96	Cell wall. Nucleus.	MT465943
*MdPAP28*	MD14G1240300	Chr14:31921701-31927798	999	332	7	37.76	5.79	Nucleus.	
*MdPAP29*	MD15G1020000	Chr15:1161626-1169626	1596	531	8	60.33	5.63	Extracell.	
*MdPAP30*	MD15G1164300	Chr15:12369682-12373858	1458	485	10	56.12	8.82	Cell wall. Nucleus.	MT465944
*MdPAP31*	MD16G1013600	Chr16:1051623-1054883	1005	334	8	37.77	4.74	Nucleus.	

**Table 2:** The hydroponic conditions of *Malus hupehensis* var. *pingyiensis* plants were used in this study.

Groups	Content of KH_2_PO_4_	Osmotic Pressure
CK	500 μM KH_2_PO_4_	0 MPa
LP	5 μM KH_2_PO_4_	0 MPa
HP	5 mM KH_2_PO_4_	0 MPa
D	500 μM KH_2_PO_4_	−0.75 MPa
DLP	5 μM KH_2_PO_4_	−0.75 MPa
DHP	5 mM KH_2_PO_4_	−0.75 MPa

## Data Availability

The original contributions presented in this study are included in the article/Supplementary Materials. Further inquiries can be directed to the corresponding author.

## Supplementary Materials

The following supporting information can be downloaded at: https://www.mdpi.com/article/10.3390/ijms26031011/s1.
