# Supplementary material for: Genome-Wide Analysis, Identification, and Transcriptional Profile of the Response to Abiotic Stress of the Purple Acid Phosphatases (PAP) Gene Family in Apple
Source: Int J Mol Sci. 2025 Jan 24;26(3):1011. doi: 10.3390/ijms26031011 (PMC11816921; doi:10.3390/ijms26031011)
Supplement: Supplementary file 1 [file ijms-26-01011-s001.zip › Table S1.pdf]

Table S1. The conservative domain analysis of purple acid phosphatases (PAPs) proteins in *Malus domestica*.

**proteins**	**PAP Conserved Motif**				
	**I**	**II**	**III**	**IV**	**V**
**MdPAP1**	ADGK	GDNIF	GNHD	VMKH	GHDH
**MdPAP2**	ADMH	GDNIF	GNHD	VMKH	GHDH
**MdPAP3**	GDLG	GDLSY	GNHE	VLMH	GHVH
**MdPAP4**	GDLG	GDLSY	GNHE	VLFH	GHVH
**MdPAP5**	GDLG	GDVTY	GNHE	AAWH	GHVH
**MdPAP6**	ADMH	GDNIF	GNHD	VFFH	GHDH
**MdPAP7**	GDLG	GDLSY	GNHE	VLMH	GHVH
**MdPAP8**	GDLG	GDVTY	GNHE	ATWH	GHVH
**MdPAP9**	GDLG	GDIVY	GNHE	FAAH	GHVH
**MdPAP10**	GDMG	GDMPY	GNHE	FAAH	GHVH
**MdPAP11**	GDMG	GDLSY	GNHE	FAAH	GHVH
**MdPAP12**	GDWG	GDNFY	GNHD	VVGH	GHDH
**MdPAP13**	HDPG	GDISY	GNHE	VMGH	GHVH
**MdPAP14**	ADMH	GDNIY	GNHD	VMKH	GHDH
**MdPAP15**	GDLG	GDLSY	PNHD	ILMH	GHVH
**MdPAP16**	GDLG	GDVTY	GNHE	ATWH	GHVH
**MdPAP17**	GDLG	GDVTY	GNRE	AVQH	GHGI
**MdPAP18**	GDLG	GDLSY	GNHE	VLFH	GHVH
**MdPAP19**	GDLG	GDLSY	GNHE	VLLH	GHVH
**MdPAP20**	GDLG	GDLSY	GNHE	VLIH	GHVH
**MdPAP21**	GDWG	GDNFY	GNHD	VVGH	GHDH
**MdPAP22**	GDWG	GDNFY	GNHD	VVGH	GHDH
**MdPAP23**	GDMG	GDISY	GNHE	VQGH	GHVH
**MdPAP24**	GDLG	GDLCY	GNHE	AAWH	GHVH
**MdPAP25**	GDLG	GDLSY	GNHE	VLLH	GHVH
**MdPAP26**	GDLG	GDLSY	GNHE	VLLH	GHVH
**MdPAP27**	GDMG	GDLTY	GNHE	FAAH	GHVH
**MdPAP28**	GDWG	GDNFY	GNHD	VVGH	GHNH
**MdPAP29**	GDLG	GDLSY	GNHE	VLMH	GHVH
**MdPAP30**	GDLG	GDLSY	GNHE	VLMH	GHVH
**MdPAP31**		GDNFY	GNHD	VVGH	GHLH
