# Supplementary material for: Genome-Wide Analysis, Identification, and Transcriptional Profile of the Response to Abiotic Stress of the Purple Acid Phosphatases (PAP) Gene Family in Apple
Source: Int J Mol Sci. 2025 Jan 24;26(3):1011. doi: 10.3390/ijms26031011 (PMC11816921; doi:10.3390/ijms26031011)
Supplement: Supplementary file 1 [file ijms-26-01011-s001.zip › Table S2.pdf]

Table S2. Primers used for cloning of *MdPAP* genes and quantitative real-time PCR analyses.

Gene name	Primers for cloning (5'–3')	Primers for qRT-PCR (5'–3')
**MdPAP1**		5' TCGGTTCTCAATCTTTACTTCC 3'
		5' GCTAATGTCTTCTTGCCTCACT 3'
**MdPAP2**	5' ATGAGTGGTACAGTGACAGTGAG 3'	5' CGGAGTTGAGGGCTCTGGTT 3'
	5' TCAATTACCGCCAACAGGTTTTC 3'	5' CCGGCAATGGTATGTGAAAGTA 3'
**MdPAP3**	5' ATGATGGCAGCGGTTACTAGGATG 3'	5' TGAAGAGTGCAGGACAGGC 3'
	5' TCAATACGTAGCACTTTCGTCC 3'	5' GATGGCATACCAAAGAGGG 3'
**MdPAP4**	5' ATGGACCCAAAGCCGATAATCACG 3'	5' CATTCAGTCTCGGTGGGATA 3'
	5' CTATGGTTCCGTAAGTATCTTCG 3'	5' CACTTTCTTCATACGGCATTT 3'
**MdPAP5**		5' CTCATTCTCGCAAACTCCTATT 3'
		5' CGATCTCATGGTTCCCTTCTAC 3'
**MdPAP6**		5' TCCGAGTTTGACTGGTGTTCT 3'
		5' TTCTTGGTCGTGGTTTCCTAA 3'
**MdPAP7**		5' ATGTCAGGTGGGATACTTGGG 3'
		5' TTGGTTTCACCGATTTCTGG 3'
**MdPAP8**		5' CGTGTTTGATTGCGGTGGTT 3'
		5' GGAAATCTGCTCGGGCTCA 3'
**MdPAP9**		5' CACCTACCGACGATAGAGAAG 3'
		5' TGAGAATAATGCGAATGAAAA 3'
**MdPAP10**		5' AACAAGGAGGGAGGTGATGGA 3'
		5' CGTGGTGCCGAGAAGTAGAGG 3'
**MdPAP11**	5' ATGGAGCAAAGTTTTCAACTTTTTG 3'	5' TGGGTTTGACGAACTTGGGC 3'
	5' CTAGGACGCAGCGGTCGTC 3'	5' AGGAGAGGCATTGATGGAGG 3'
**MdPAP12**		5' GGATGTGGAGTTGGCATTGAA 3'
		5' GGATTGGGAGAAGCTGGTTTG 3'
**MdPAP13**		5' GAAAAACTACCTCTTCTCTGCC 3'
		5' ATGAAATGATAATGAACCACCA 3'
**MdPAP14**	5' ATGAAGAGGGACAGGGAGGTG 3'	5' ATTACTCTACGGTTCCCTCCATA 3'
	5' TCAGTTACCGCCAACAGGTTTC 3'	5' ACACTGCCTTCACATCTCCTGCT 3'
**MdPAP15**	5' ATGTATTTCAATACCAAATACTACT 3'	5' GCTGTACTGTTTGTTGGGGAC 3'
	5' TCATGATTTGGCAGTTGTGG 3'	5' GCAAGGCTTAAAAGGTTTGGT 3'
**MdPAP16**	5' ATGGCATTTTCTTCGTGCTCC 3'	5' TCGCTATCCGCTGATTACGACTCC 3'
	5' TTAGAGGTCAGCAAGCCAAC 3'	5' AACGAACAACGCTTGCCACACTTT 3'
**MdPAP17**		5' CCACAGGGGTTGAAACTGAATA 3'
		5' CGAGGACCAGAAACTGGGATAG 3'
**MdPAP18**	5' ATGGACCCCAAGCAGATAATCAC 3'	5' CAAGTATAAAAGCCCATCACCTG 3'
	5' CTATGGTTGGATGAGTATCTTCCTT 3'	5' CAACGCATCCCGAACTAACCAAC 3'
**MdPAP19**		5' TTGTGGAGTATGGAAAGGAATC 3'
		5' TATAGAAGTATGTAGTGGCAGG 3'
**MdPAP20**	5' ATGGCGATCAAACGCCGG 3'	5' GGGACTGGTTCTCGCTGTTTTTA 3'
	5' TTACTTCTTGCAGGCAGGATCAG 3'	5' GATCCATGTCACCCTCATTTTGT 3'
**MdPAP21**		5' TCTATGCACCCTGTCATCTTTT 3'
		5' GATTGGTTGTAGGCTCCTCTTC 3'
**MdPAP22**		5' GTCACTTTGTTTGGTTGCTTGC 3'
		5' ATTGGTTGTATTGTCCCCGTCT 3'
**MdPAP23**	5' ATGGCCGTTCCTTCAACAACC 3'	5' CCCACAGCCAGAGCGGTCTTTAT 3'
	5' TCAGGTTTCCTCACTCTTCACAG 3'	5' CGTGTCGTGCACCTTCCCATCAT 3'
**MdPAP24**		5' TGGGAGTGAGGTGAGGTATGGGG 3'
		5' CTCTGGTAAAGTTTCAAGGGCGT 3'

**MdPAP25**		5' TCCACCTCCAACTTGTACTACTC 3'
		5' TCATACTCTCCCCTTCACCTTTA 3'
**MdPAP26**		5' TCCACCGTCTCGCAAGAACCTCTA 3'
		5' TCACCCTCATTTTGTCCTCGCCTA 3'
**MdPAP27**	5' ATGGCGCAAAGTTTTCAACTG 3'	5' GCGATTGTGGTGATTCTGGGATTG 3'
	5' CTACGACGCAGCGGTAG 3'	5' GGATGAGAGGTTGATGGTGGCTTT 3'
**MdPAP28**		5' TTTTTCTTGATCCTCAGTTTTGCC 3'
		5' ATCTCCTCTCCTTCCCCAGTCCCC 3'
**MdPAP29**		5' TCTCTTCACCCAGCGCCAACACCT 3'
		5' GCTCATCCCTGCCTTCCTCATCCC 3'
**MdPAP30**	5' ATGACGGCAGTGGTTACTAG 3'	5' TCCTTCGTCTTGTTGAGCCTTTTG 3'
	5' TCAATACATAGCAATTTCGTCCATT 3'	5' GTTGTGGAGCATTGTAACCCTTCG 3'
**MdPAP31**		5' GGTCAAGTATTCTGTTTCCTGCT 3'
		5' TCCATTATCGTAAAAATTATCGC 3'
**Action**		5' AACAATGCTAGGGAACACGGCTCT 3'
		5' ACAGGAAGTAGAAGATGGCGGACA 3'
