# Supplementary figures and images for: Genome-Wide Analysis, Identification, and Transcriptional Profile of the Response to Abiotic Stress of the Purple Acid Phosphatases (PAP) Gene Family in Apple
Source: Int J Mol Sci. 2025 Jan 24;26(3):1011. doi: 10.3390/ijms26031011 (PMC11816921; doi:10.3390/ijms26031011)

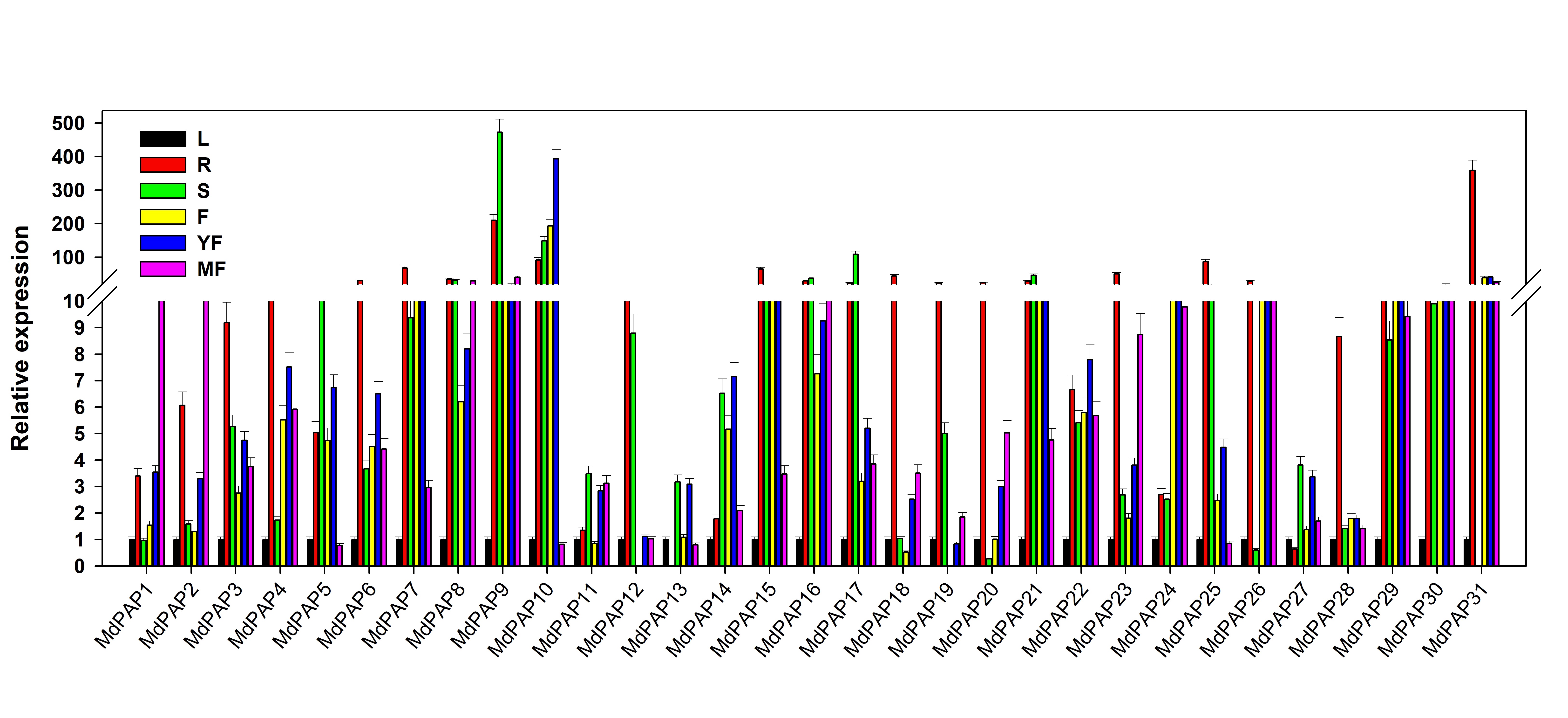

Supplement: Supplementary file 1 [file ijms-26-01011-s001.zip › Fig S1.JPG]

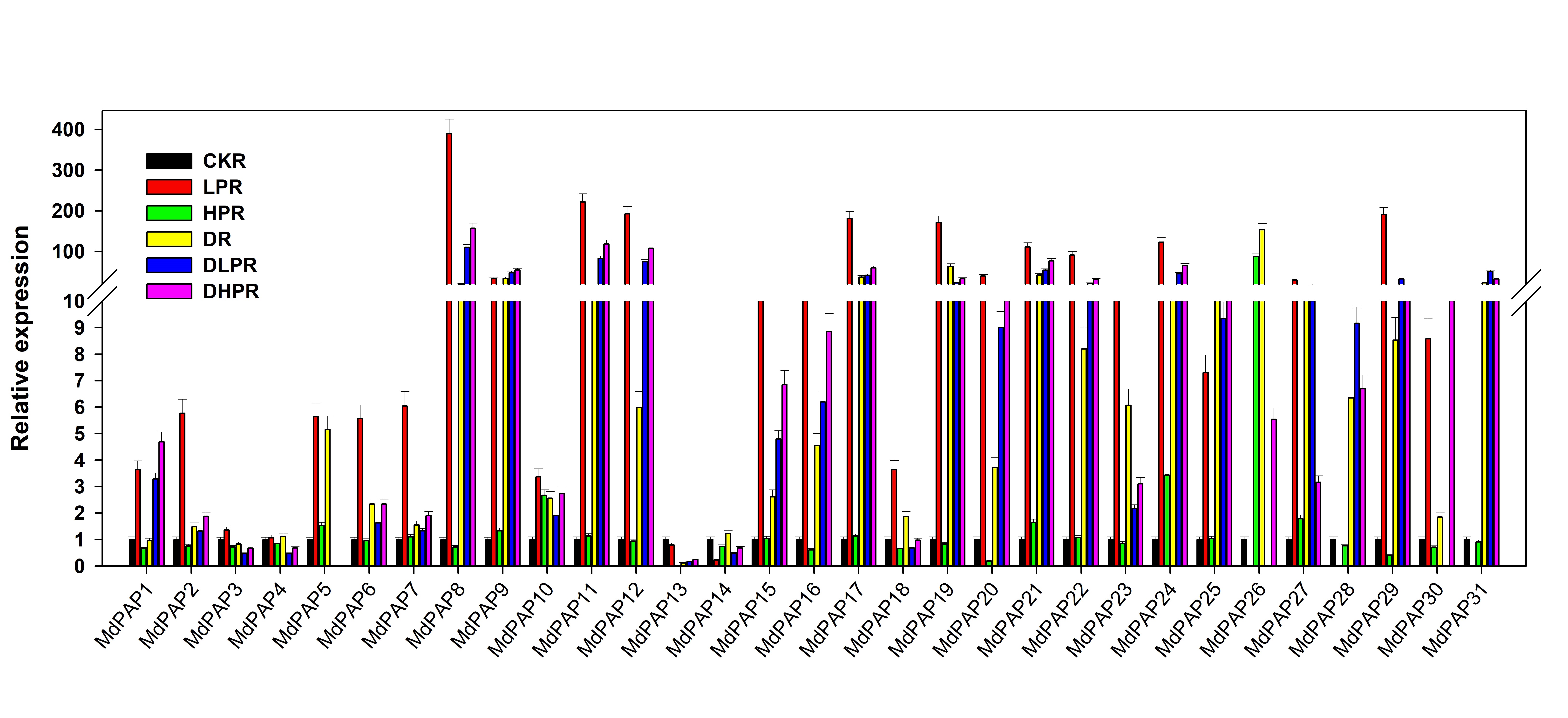

Supplement: Supplementary file 1 [file ijms-26-01011-s001.zip › Fig S2.JPG]

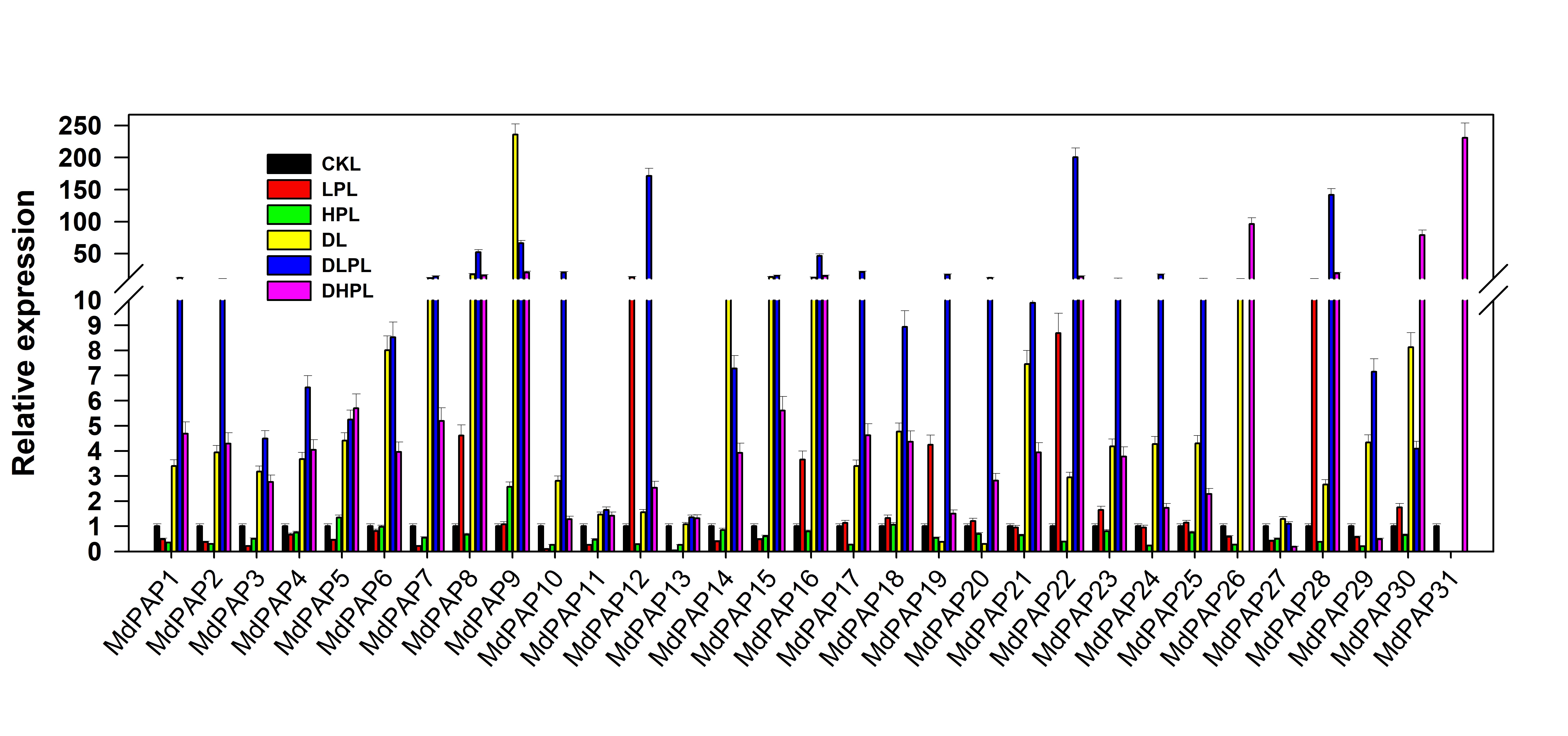

Supplement: Supplementary file 1 [file ijms-26-01011-s001.zip › Fig S3.JPG]
